# One-year timeline kinetics of cytokine-mediated cellular immunity in dogs vaccinated against visceral leishmaniasis

**DOI:** 10.1186/s12917-015-0397-6

**Published:** 2015-04-11

**Authors:** Christiane Costa-Pereira, Marcela L Moreira, Rodrigo P Soares, Bruno H Marteleto, Vitor M Ribeiro, Michelle H França-Dias, Ludmila M Cardoso, Kelvinson F Viana, Rodolfo C Giunchetti, Olindo A Martins-Filho, Márcio S S Araújo

**Affiliations:** Laboratório de Biomarcadores de Diagnóstico e Monitoração, Centro de Pesquisas René Rachou/FIOCRUZ – MG, Av. Augusto de Lima, 1715, 30190-002 Belo Horizonte, MG Brazil; Laboratório de Biologia das Interações Celulares, Departamento de Morfologia, Universidade Federal de Minas Gerais, Av. Antônio Carlos, 6627, 31270-901 Belo Horizonte, MG Brazil; Clínica Veterinária Santo Agostinho, Avenida Amazonas, 2218, 30180-00 Belo Horizonte, MG Brazil

**Keywords:** Canine visceral leishmaniasis, Vaccine, Leishmune®, Cytokines

## Abstract

**Background:**

The main control strategy for visceral leishmaniasis in Brazil has been based on the elimination of seropositive dogs, although this is not widely accepted. In this context, the use of a long-lasting protective vaccine against canine visceral leishmaniasis (CVL) has been highly expected. The aim of this work was to determine the timeline kinetics of the cytokine microenvironment derived from circulating leukocytes as supportive immunological biomarkers triggered by Leishmune® vaccine. Cross-sectional kinetic analysis of cellular immunity cytokines was carried out at three times (1, 6 and 12 months) after primovaccination with Leishmune®. *In vitro* short-term whole blood cultures were stimulated with *Leishmania infantum* soluble antigen (SLAg). The secreted cytokine signatures and their major sources were determined.

**Results:**

At six months after vaccination, Leishmune® induced an increase in IL-8, IFN-γ, IL-17a and TNF-α levels and a decrease in IL-10. Cytokine signature analysis revealed a shift in the microenvironment towards a pro-inflammatory profile mediated by IL-8 and IFN-γ. Both, CD4^+^ (↑TNF-α^+^ and ↑IFN-γ ^+^) and CD8^+^ (↑IL-17a and ↓IL-4) T-cells contributed to the acquired immune responses observed after stimulation with SLAg.

**Conclusions:**

The changes observed in the cytokine profile suggested that Leishmune® was able to induce an effective response at six months after primovaccination. After one year, it returned to baseline suggesting the need of additional boosting.

## Background

Visceral leishmaniasis (VL), caused by *Leishmania infantum* (syn. *Leishmania chagasi*), is the most severe and fatal form of leishmaniasis. Canine visceral leishmaniasis (CVL) is a serious public health problem in the Americas, Mediterranean region, Africa, Asia and Europe [1]. Dogs are very susceptible to infection and the most important domestic hosts [2,3]. Euthanasia of seropositive dogs is recommended, but it did not effectively decrease the number of canine and human cases in Brazil [4]. Although chemotherapy may reduce or eliminate clinical signs in sick dogs, parasitological cure is not achieved [5]. In fact, a large proportion of infected dogs may become subclinically infected after chemotherapy, but some of them can still transmit the parasite to the sand fly vector. The use of topical insecticides and impregnated collars, as well as vaccines, is difficult to implement in a nationwide control program [6]. In the absence of other successful strategies, vaccine development against CVL has been quoted as one more promising control measure [7]. There is a clear consensus that CVL immunoprofilaxis may also have a positive impact on the human leishmaniasis transmission. However, although some available vaccines decrease infectiousness of the disease for dogs, they still allowed sand fly infection [8].

The course of *L. infantum* infection in dogs depends on the host immune response, persistence and multiplication of the parasite. The innate and adaptive immunity components are engaged in a wide range of complex interactions. The initial steps in the innate immune compartment are important for a successful development of an acquired immune response [9,10]. Cytokines play a decisive role during *L. infantum* infection [11]. Cellular immune responses mediated by pro-inflammatory cytokines (IFN-γ and TNF-α, were predominant in subclinically infected dogs, suggesting their putative role for protection against the disease. On the other hand, regulatory cytokines (IL-4 and IL-10) seemed to be associated with disease progression and severity [12]. However, there is a consensus that a pro-inflammatory cellular immunity plays a relevant role in the protective events during CVL [12-14].

Many efforts have been made by several groups in order to develop a vaccine against CVL [15-17]. In Brazil, the vaccine Leishmune® (Pfizer-Zoetis) was used for many years. Nowadays, only one commercially available vaccine is in use (Leish-Tec) (Hertape Calier). Leishmune® is composed of a glycoproteic complex, fucose–mannose ligand (FML), and Leish-Tec consists of the A2 recombinant protein in saponin. Field studies in Brazil endemic areas demonstrated that Leishmune® exhibited 76-80% efficacy [18-20]. Leish-Tec was also a potent immunobiological tool to prevent CVL, inducing high levels of IgG2 and IFN-γ with concomitant decrease of IL-10 [21].

Here, the one-year timeline kinetics of the pro-inflammatory and regulatory cytokines was evaluated in Leishmune® vaccinated dogs. The cytokine profile produced by circulating leukocytes after short-term *in vitro* stimulation with LSAg enabled the establishment of supportive immunological biomarkers after primovaccination.

## Methods

### Ethics statement

The study protocol was approved by the Ethical Committee for the Use of Experimental Animals (CEUA) of the Fundação Oswaldo Cruz (FIOCRUZ - PROTOCOL No. P-71/11-3).

#### Dogs

In this work, 40 dogs of different breeds (18 males and 22 females), with ages ranging from 8 months to 8 years, were selected in veterinary clinics located in Belo Horizonte, Minas Gerais, Brazil and Informed written consent was obtained from owners of all dogs. All dogs were evaluated during the course of the experiments by their respective Veterinary Doctors. Only healthy dogs from the same area with negative serology (ELISA and Indirect Immunofluorescence - IIF) for CVL and regular anti-helmintic treatment were included in the study. Leishmune® primovaccination was confirmed by immunization cards or in the clinical record files at the veterinary clinics. This is a cross-sectional study structured in three groups of 10 animals each, categorized according time after Leishmune® primovaccination (T1, T6 and T12 stand for one, six and twelve months post-vaccination, respectively). All dogs had received subcutaneously the complete immunization protocol proposed by the Leishmune® manufacturer (Pfizer-Zoetis, Campinas, and São Paulo, Brazil). This consisted of three shots with 21 days interval. A group of ten seronegative non-vaccinated conscripts was included as control (T0) to evaluate the pro and anti-inflammatory basal levels of cytokines.

#### Leishmania infantum soluble antigen (SLAg)

*Leishmania infantum* promastigote forms (MHOM/BR/1970/BH46) were grown in liver infusion tryptose medium (LIT), supplemented with 10% of fetal bovine serum at 24°C. SLAg production used stationary-phase parasites (7 days of growth) as described [22]. Final protein concentration was adjusted to 1 mg/mL. Aliquots were stored at -70°C prior to short-term *in vitro* stimulation.

#### Short-term whole blood culture in vitro

Five mL of whole peripheral blood were collected from each dog in heparinized vacuum tubes. *In vitro* short-term cultures were performed as described by Silva *et al.* [23] and whole blood leukocyte counts were determined by automated hematology analyzer (Advia60, Bayer Diagnostics, Tarrytown, NY, USA). Aliquots of 500 μL of heparinized peripheral blood (containing 5-7×10^6^ whole blood leukocytes) were transferred to lithium heparin Plasma Separator Tubes (Vacutainer® PST tubes, BD, Franklin Lakes, NJ, USA) and incubated for 48 hours at 37°C, 5% CO_2_, in the presence of 475 μL of RPMI 1640 medium (GIBCO, Grand Island, NY). One tube, referred as Ag-Stimulated Culture (SLAg), received 25 μL of *L. infantum* soluble antigen (SLAg at 1 mg/mL, final concentration 25 μg/mL). A second tube, Control Culture (CC), received 25 μL of RPMI. Finally, as internal positive control, was incubated with phorbol myristate acetate (PMA; 25 ng/mL) and ionomycin (1 μg/mL) in RPMI. The cultures were incubated at 5% CO_2_, 37°C for 48 hours.

#### Analysis of peripheral leukocyte secreted cytokines by enzyme-linked immune sorbent assay (ELISA)

Following short-term whole blood culture *in vitro*, the tubes were centrifuged 1,400 *g* for 10 minutes, and the supernatant stored at -80°C in 100 μL aliquots for soluble cytokines quantification by ELISA.

Soluble cytokine levels were determined by DuoSet enzyme-linked immunosorbent assay (ELISA) to quantify IL-8 (anti-canine IL-8, catalog number: DY1608); TNF-α (anti-canine TNF-α /TNFSF1A immunoassay; catalog number: DY1507); IFN-γ (anti-canine IFN-γ, catalog number: DY781B) and IL-10 (anti-canine IL-10, catalog number: DY735). Home-standardized ELISA was carried out to quantify IL-4, using monoclonal anti-canine IL-4 antibody (catalog number: MAB7541) as capture antibody; recombinant canine IL-4 (catalog number: 754CL) for obtaining the standard curve and biotinylated anti-canine IL-4 antibody (catalog number: BAF754), avidine peroxydase (R&D Systems, DY998), and substrate solution (1:1 mixture of H_2_O_2_ and tetramethyl-benzidine, product code 50-76-4, lot. no. RB49).

Briefly, the ELISA assays were carried out by adding 25 μL of PBS-diluted monoclonal anti-cytokine to 96 well plates (COSTA®, Washington, DC, USA), followed by overnight incubation at room temperature. After four wash steps with PBS-Tween 20 buffer, blocking procedures were carry out for 1 h with 0.1% of bovine serum albumin (BSA) and 0.05% sodium azide in PBS. Following four wash steps with PBS-Tween 20 buffer, 25 μL of culture supernatant were added to specific wells. Alongside, 25 μL of serial diluted recombinant cytokine were used to establish the standard curve. The plates were incubated for 2 h at room temperature, washed twice with PBS-Tween 20 and 25 μL of biotinylated anti-cytokine antibody were added to each well. After two wash steps with PBS-Tween 20, 25 μL of avidine peroxydase diluted in PBS – 0.1%BSA were added to each well and incubated for 30 min at room temperature. After washing steps with PBS, 25 μL of substrate solution (H_2_O_2_ and tetramethylbenzidine, 1:1) were added and after 10 minutes 25 μL of stopping solution (H_2_SO_4_, 1 M) used as a final reagent. The optical density was determined by automatic Absorbance Microplate Reader (Biotek, EL800, Winosski, VT) at a wavelength of 450 nm. Minimum sensitivity of ELISA were IL-8 (15.6 pg/mL), TNF-α 15.6 pg/mL), IFN-γ (31.2 pg/mL) and IL-10 (31.2 pg/mL) as provided by the manufacturer.

#### Immunophenotypic and intracytoplasmic cytokine staining

**Figure 1:**
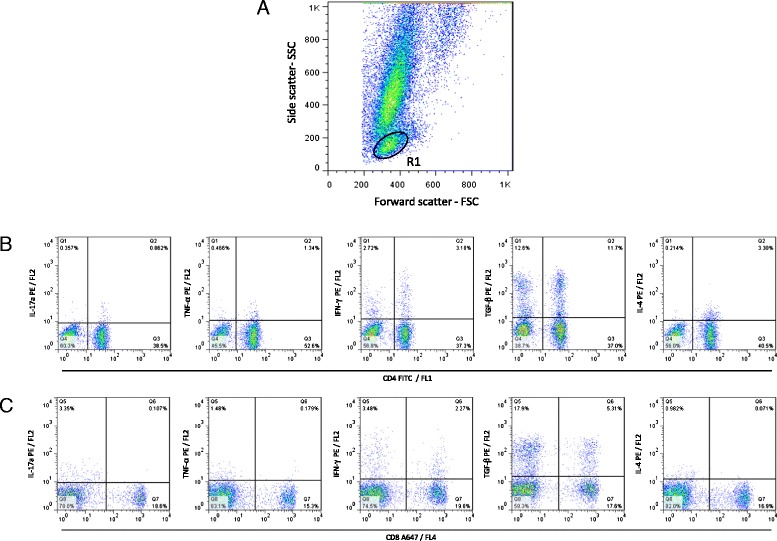
Representative dot plots illustrating the analysis of intracellular cytokine profile in T-cell subsets. **(A)** Pseudocolor plot distribution of short-term *in vitro* cultured (control or SLA-Ag stimulated) canine whole blood sample according to cell size (Forward scatter - FSC) and granularity (Side scatter- SSC) used for lymphocyte gate selection (R1) of FSC^Low^SSC^Low^ events. **(B)** Pseudocolor plots representing cytokines + (IL-17, TNF-α, IFN-γ, TGF-β and IL-4) CD4^+^ cells within gated lymphocytes and **(C)** Pseudocolor plots representing cytokines + (IL-17, TNF-α, IFN-γ, TGF-β and IL-4) CD8^+^ cells within gated lymphocytes. The frequency of cytokines^+^ T-cells subsets were calculated by quadrant statistics approach and first reported as percentage of gated lymphocytes prior to the calculation of the SLAg/Control indexes.

After short-term whole blood culture *in vitro*, 10 μL of Brefeldin A (BFA; Sigma Chemical Company, St. Louis, MO) at 1 mg/mL was added to each tube and cultures were submitted to an additional incubation for four hours at 5% CO_2_, 37°C. Then, 200 μL of ethylene diamine tetra acetic acid – EDTA (Sigma, St Louis, USA) were added to each culture tube (2 mM final concentration). After 10 minutes of incubation at room temperature, cultured whole blood samples were washed with PBS supplemented with 0.5% bovine serum albumin and 0.1% sodium azide (Sigma, St. Louis, USA). The cells were resuspended in 500 μL of PBS-0.5% BSA. Aliquots of 100 μL were stained with anti-cell surface monoclonal antibodies (anti-CD4-FITC and anti-CD8-A647, Table 1) for 30 minutes at room temperature. After membrane staining, erythrocyte lysis, and leukocytes fixation, the cell suspension was permeabilized with PBS-0.05% BSA buffer supplemented with 0.5% saponin. Aliquots of 50 μL were incubated in the presence of phycoerythrin fluorochrome (PE)-labeled anti-cytokines mAbs (IL-17a, TNF-α, IFN-γ, TGF-β and IL-4 - Table 1), for 30 minutes at room temperature, in the dark. After intracytoplasmic cytokine staining, the leucocytes were washed with PBS-0.05% BSA and fixed in FACS fixing solution (10 g/L paraformaldehyde, 10.2 g/L sodium cacodylate, and 6.63 g/L sodium chloride, pH 7.2). Flow cytometric measurements were performed on a FACScalibur instrument (Becton Dickson - BD, USA) interfaced to an apple G3 FACStation. The Cell-Quest™ software package provided by the manufacturer (Franklin Lakes, NJ, USA) was used for data acquisition and analysis. A total of 30,000 events were acquired for each preparation. The frequency of CD4^+^ and CD8^+^ T-cells expressing intracytoplasmic cytokines (IL-17a, TNF-α, IFN-γ, TGF-β and IL-4) was determined following the conventional strategy analysis. This analysis consisted of selecting the population of interest, based in morphometric aspects, through punctual distribution of size (forward scatter- FSC) and granularity (side scatter - SSC) graphs. After the selection of the interest region R1 containing FSC^Low^ SSC^Low^ phenotype cells, graphs of density plot distribution of CD4/FL1 or CD8/FL4 *versus* IL-17a/FL2, TNF-α/FL2, IFN-γ/FL2, TGF-β/FL2 or IL-4/FL2 were made to determine the percentage of IL-17a^+^, TNF-α^+^, IFN-γ^+^, TGF-β^+^ and IL-4^+^ cells inside the previously selected lymphocytes (Figure 1). Final data was expressed as cytokine indexes, calculated by dividing the percentage of cytokine positive cells observed in the SLAg-stimulated culture by the one observed in paired control unstimulated culture (SLAg/CC ratio).

**Table 1:** **Monoclonal antibodies used for immunophenotyping assays intracytoplasmic detection of cytokines** ***in vitro***

**Marker**	**Host**	**Clone**	**Fluorochrome**	**Manufacturer**
Canine Anti-CD4	Rat	YKIX302.9	FITC	Serotec
Canine Anti-CD8	Rat	YCATE55.9	A647	Serotec
Human Anti-IL17-a	mouse	64DEC17	R-PE	BD Pharmingen
Human Anti-TNF-α	mouse	MAb11	R-PE	BD Pharmingen
Bovine Anti-IFN-γ	mouse	CC302	R-PE	Serotec
Human Anti-TGF-β	mouse	TB21	R-PE	IQ Products
Bovine Anti-IL 4	mouse	CC303	R-PE	Serotec

### Comparative data analysis

#### Conventional statistical analysis

The statistical analysis was performed using the software GraphPad Prism 5.03. For the data presenting a parametric distribution, for example, intracytoplasmatic cytokines, variance analysis (ANOVA) was used, followed by Tukey test. For the non-parametric distribution, for example, secreted cytokines in the supernatant, it was performed the Kruskal-Wallis test, followed by Dunns test. Differences were considered significant at p ≤ 0.05.

#### Comparative analysis of cytokines signatures secreted by peripheral leucocytes

**Figure 2:**
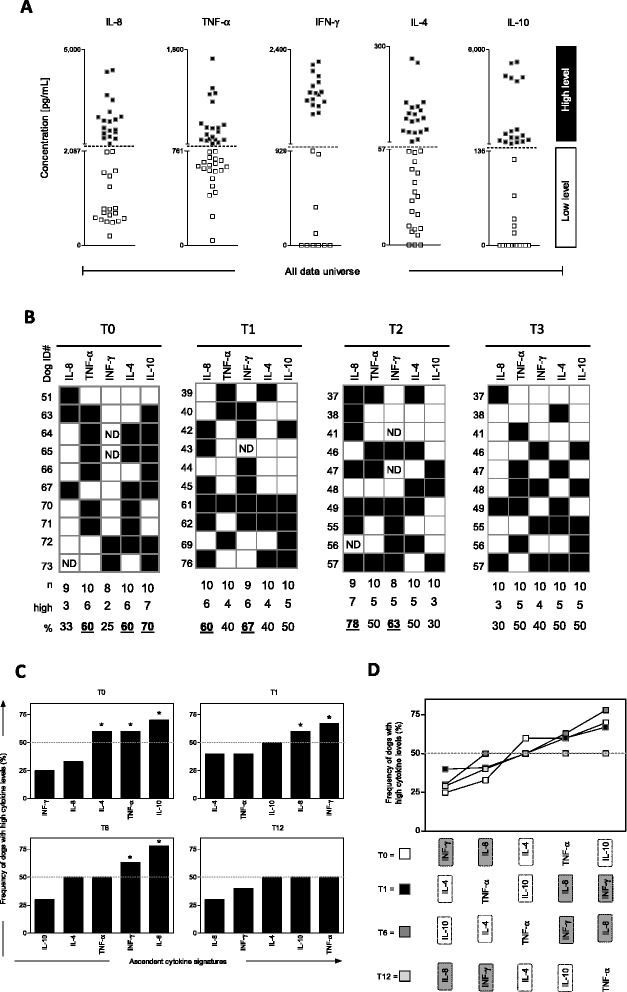
Signature analysis of secreted cytokine by peripheral blood leukocytes afterin vitrostimulation withLeishmania infantum soluble antigens (SLAg). **(A)** Establishment of the global median cut-off edges for each cytokine (IL-8, TNF-α, IFN-γ, IL-4 and IL-10) used to segregate dogs as they present “Low” () or “High” () cytokine levels. **(B)** Gray-scale diagrams used to compile the frequency (%) of high cytokine producers. **(C)** Ascendant cytokine signatures were assembled for each time after vaccination (T0, T1, T6 and T12). The frequencies of high producers were considered relevant (*) when the percentage was confined over the 50^th^ percentile (doted lines). **(D)** Comparative analysis of cytokine signatures were used to identify relevant differences amongst Leishmune® vaccinated dogs at one (T1 = black rectangle), six (T6 = dark grey rectangle) and twelve (T12 = light grey rectangle) months post-vaccination compared to unvaccinated dogs (T0 = white rectangle). Gray scale rectangles were used to highlight the shift in the overall profile of pro-inflammatory and regulatory cytokines on each time after immunization. Relevant differences (shift of frequencies across the 50^th^ percentile cut-off edge) were highlighted by gray background.

Cytokine signatures were compared to characterize the pattern of secreted cytokines from each animal vaccinated as suggested [23,24]. Briefly, the “global median” value for each secreted cytokine was calculated by taking the whole universe of data (T0 + T1 + T6 + T12). The “global median” was used as the cut off to tag each dog as they display “Low” or “High” levels of secreted cytokines in the culture supernatant as follows: (IL-8 = 2,087 pg/mL; TNF-α = 761 pg/mL; IFN-γ = 929 pg/mL; IL-4 = 57 pg/mL and IL-10 = 135 pg/mL) (Figure 2A). Following the assembling of dogs categorized as “Low” or “High” cytokines producers, the frequency was calculated using gray-scale diagrams for each timeline (T0, T1, T6 and T12) (Figure 2B). The ascendant frequency of high cytokine for each timeline was assessed to generate the T0, T1, T6 and T12 cytokine signatures (bar charts) (Figure 2C). Relevant frequencies of “high cytokine producers” were considered when the percentage was above the 50^th^ percentile. In a final step, the cytokine signatures were converted from the bar chart to line curves for overlaid comparative analysis amongst each timeline (T0, T1, T6 and T12). Relevant differences (shift of frequencies across the 50^th^ percentile cut-off edge) were highlighted by gray background (Figure 2D).

## Results

### Timeline kinetics of cytokines secreted by peripheral blood leukocytes after in vitro stimulation

**Figure 3:**
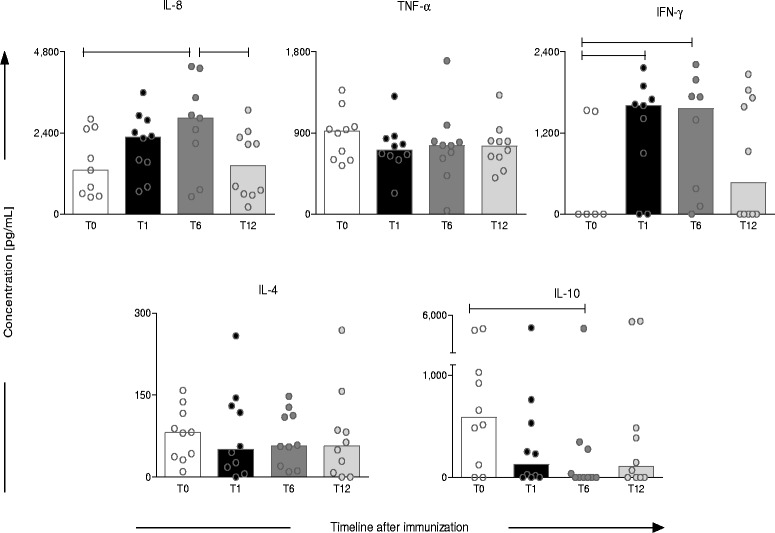
Profile of cytokines secreted by peripheral blood leukocytes afterin vitrostimulation withLeishmania infantum soluble antigens (SLAg). Short-term whole blood cultures were performed to characterize the cytokine profile secreted by circulating leukocytes from Leishmune® vaccinated dogs at one (T1 = black rectangle), six (T6 = dark grey rectangle) and twelve (T12 = light grey rectangle) months post-vaccination compared to unvaccinated dogs (T0 = white rectangle). Data are reported as cytokine levels in culture supernatants in picograms/mL (pg/mL). Results are expressed as median values over scattering distribution of cytokine levels. Significant differences at p ≤ 0.05 are highlighted by connecting line.

After stimulation with SLAg, a peak of leukocyte-secreted pro-inflammatory cytokines (IL-8 an IFN-γ) was observed at T6. This was concomitant with a decrease of IL-10 compared to non-immunized dogs. Moreover, it was noticed that IL-8 levels return to baseline at T12. More importantly, IFN-γ, a relevant immunological biomarker, was significantly increased at T1, re-enforcing the ability of Leishmune® to trigger a protection pattern in primovaccinees. As expected, there is some basal production of cytokines by the dogs at T0 (Figure 3).

### Cytokine signature analysis further demonstrated that Leishmune® vaccination shifted the immune response towards a pro-inflammatory profile

After cytokine signature analysis (Figure 2), the frequency of high producers was determined for each experimental group of animals. This qualitative data re-emphasized the supportive immune protection biomarkers observed after Leishmune® vaccination. A shift towards pro-inflammatory cytokines (IFN-γ and IL-8) was observed from T0 to T1 and sustained at T6. Concomitant decrease of regulatory cytokines (IL-4 and IL-10) was also observed at T1 and T6 compared to T0. Moreover, it was important to notice that at T12, the cytokine signatures returned to baseline profile similar to T0, with enhanced frequency of dogs presenting high levels of IL-10 and IL-4.

### Intracytoplasmic cytokine indexes in peripheral blood leukocytes from Leishmune® vaccinated dogs after *in vitro* stimulation with SLAg

**Figure 4:**
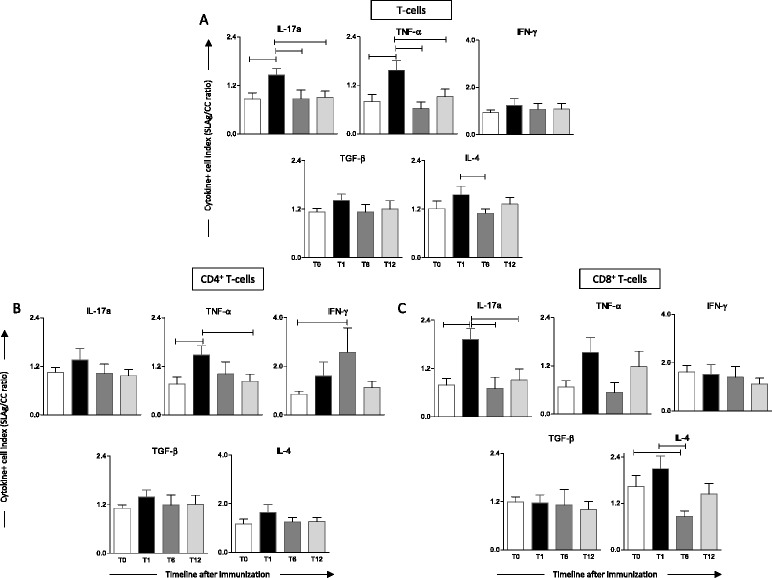
Intracytoplasmic cytokine indexes in peripheral blood leukocytes afterin vitrostimulation withLeishmania infantumsoluble antigens (SLAg). Cytokine indexes were calculated as the proportion of cytokine^+^ cells observed in SLAg-stimulated cultures divided by the control culture (SLAg/CC ratio) for Leishmune® vaccinated dogs at one (T1 = black rectangle), six (T6 = dark grey rectangle) and twelve (T12 = light grey rectangle) months post-vaccination as compared to unvaccinated dogs (T0 = white rectangle). Data are reported as cytokine indexes (SLAg/CC ratio) in lymphocytes (**A)** T-cells **(B)** CD4^+^ T-cells **(C)** CD8^+^ T-cells. Results are expressed as mean values of cytokine indexes ± standard error. Significant differences at p ≤ 0.05 are highlighted by connecting line.

In order to identify the major cytokine sources induced in peripheral blood leukocytes after *in vitro* stimulation, each dog cytokine indexes observed for T-cells and T-cell subsets were calculated (Figure 4). A clear peak of IL-17a, TNF-α derived from T-cells at T1 was observed compared to other groups. Moreover, a decreased index of IL-4 was observed at T6 compared to T1 (Figure 4A). Analysis of CD4^+^ T-cells demonstrated a peak of TNF-α production at T1 compared to unvaccinated dogs. This cytokine returned to baseline at T12 (Figure 4B). The analysis of IFN-γ further demonstrated a peak at T6 (Figure 4B). Analysis of CD8^+^ T-cells revealed a clear peak of IL-17a at T1 compared to other groups and a decrease in the IL-4 index at T6 compared to T1 and T0 (Figure 4C). Together, these findings showed that T-cells, both CD4^+^ and CD8^+^ subsets are relevant sources of pro-inflammatory cytokines demonstrating the ability of Leishmune® to trigger a protective immunological pattern in primovaccinees.

## Discussion

Previous reports have demonstrated the long lasting protection against CVL using Leishmune® in endemic areas [19]. This investigation evaluated the kinetics of the pro-inflammatory and regulatory cellular immune response in Leishmune® vaccinated dogs in a period of one year.

The immunocompetent host is able activate innate and acquired inflammatory mechanisms responsible for disease protection. In dogs, the cellular immune responses mediated by phagocytes and T-cells, primarily involving pro-inflammatory mediators (TNF-α, IL-6, IL-12 and IFN-γ) and toxic oxygen intermediates are relevant in this context. On the other hand, an impaired cellular response is consistent with susceptibility [14,25]. Here, we have evaluated by a cross-sectional investigation, the cytokine profile triggered by Leishmune®. The timeline kinetics of major pro-inflammatory and regulatory cytokines was determined over a one year period. The data observed would provide supportive insights to the animal health authorities regarding the vaccination guidelines currently recommended for the Leishmune® vaccine.

Our data demonstrated that a clear increase in IL-8 and IFN-γ in the group of Leishmune® vaccinated dogs at 6 months post-vaccination. In fact, IFN-γ increased in the first month after vaccination compared to non-vaccinated dogs. However, the increase of IL-8 and IFN-γ did not last after 12 months, showing similar levels to non-vaccinated dogs. On the other hand, a decrease in IL-10 was observed at six months after vaccination, returning to similar levels of the non-vaccinated dogs at T12.IL-8 is essential for neutrophil activity during the early events of the immune response against *Leishmania* [26]. In our previous studies we have demonstrated that Leishmune® vaccination induced an increase in IFN-γ production by CD4^+^ T-cells [22,27]. The increase of those two cytokines in vaccinated dogs is consistent with the ability of Leishmune® to trigger an immune response. In this context, they could be classified as immuneprotection biomarkers, since they returned to baseline at T12. The regulatory cytokine IL-10 is known to modulate the pro-inflammatory immune response, inhibiting macrophages and promoting intracellular infection. Its low levels at T6 could support a pro-inflammatory immune profile, enabling parasite elimination [28].

In this study, one innovative tool [23,24] was also used to evaluate the cytokine profiles triggered by Leishmune®. Cytokine signature analysis revealed a shift in the cytokine milieu after SLAg stimulation. Similar to our previous observations, a shift towards a pro-inflammatory profile, especially mediated by IL-8 and IFN-γ was observed at T1 and T6. Also, signature analysis showed a clear decreased in those cytokines at T12 with a simultaneous increase in the frequency of regulatory cytokines, at similar levels to non-vaccinated dogs. This qualitative approach also re-enforced the use of some of those leukocyte secreted cytokines as supportive immuneprotection biomarkers. Those may be useful to follow vaccination protocols after SLAg stimulation.

*Leishmania* elimination by phagocytes is a crucial step for infection control and several reports have demonstrated that their effective and persistent activation are controlled by cytokines derived from the adaptive immunity [29,30]. In this context, it is likely to hypothetize that the post vaccination memory mediated by adaptive immunity cells may be important for an effective activity of the innate immune mechanisms. It has been proposed that CD4^+^ T-cells play a decisive role in this process through the production of cytokines and effectors mechanisms [31]. Consistent with those observations, the analysis of the intracytoplasmic cytokine profiles in T-cell subsets supported these findings. Leishmune® vaccinated dogs showed increased indexes of IL-17a and TNF-α early (one month) after primovaccination. This is similar to previous studies showing that those cytokines were related to a CVL resistance phenotype [32,33]. The analysis of T-cell subpopulations further demonstrated that CD4^+^ T-cells were relevant sources of TNF-α and IFN-γ. At the same time, CD8^+^ T-cells were an important source of IL-17a and responsible for IL-4decrease. The latter is associated with a regulatory response modulating the pro-inflammatory profile, inhibiting macrophage activity and promoting intracellular infection [28]. Our results also showed an important increase of IFN-γ in CD4^+^ T-cells after *in vitro* stimulation with SLAg. These results were similar to those observed by *L. infantum* infected macrophages from dogs immunized with the vaccine LiESAp (constituted of purified excreted antigen of *L. infantum* promastigotes plus muramyl dipeptide as adjuvant). Additionally, their co-cultivation with total lymphocytes results in higher levels of IFN-γ [34,35]. However, our data showed a clear contribution of both CD4^+^ and CD8^+^ lymphocytes for the acquired immune responses after stimulation with SLAg.

## Conclusions

The data observed showed that Leishmune® was able to trigger changes in the immune response profiles in vaccinated dogs especially after 6 months. Although it provided relevant information regarding Leishmune® potential and immunization time, one of the limitations of this study relies on the genetic variability of the dogs. Although they had different characteristics (sex, age and breed), it was clearly demonstrated that Leishmune® vaccination was able to shift the immune response towards a pro-inflammatory profile. However, this was important to validate the vaccine status in the real context. The important finding that all biomarkers (IL-8, TNF-α, IFN-γ, IL-17a, IL-4 and IL-10) returned to baseline after one year post vaccination re-enforced the need of boosting doses. Also, it suggests their putative use as immune protection biomarkers for vaccines evaluation. The continuity of this and other studies may help to generate information to understand the immune response mechanisms during CVL immunoprophylaxis.

## Abbreviations

BFA: Brefeldin A
CC: Control culture
CVL: Canine visceral leishmaniasis
EDTA: Ethylene diamine tetra acetic acid
ELISA: Enzyme linked immune sorbent *assay*
IFN-γ: Interferon gama
IgG2: Immunoglobulin G2
IIF: Indirect immunofluorescence
IL-4: Interleukin 4
IL-8: Interleukin 8
IL-10: Interleukin 10
IL-17a: Interleukin 17a
LIT: Infusion tryptose medium
PBS: Phosphate buffered saline
PMA: Phorbol myristate acetate
SLAg: *Leishmania infantum* soluble antigens
TGF-β: Transforming growth factor beta
TNF-α: Tumor necrosis factor-alpha
T0: Group non-vaccinated
T1: Group one month post-vaccination
T6: Group six months post-vaccination
T12: Group twelve months post-vaccination
VL: Visceral leishmaniasis
